# Supplementary material for: RNA structure prediction using positive and negative evolutionary information
Source: PLoS Comput Biol. 2020 Oct 30;16(10):e1008387. doi: 10.1371/journal.pcbi.1008387 (PMC7657543; doi:10.1371/journal.pcbi.1008387)

input multiple alignment:

```
[structure]  . : : < < < _ _ _ _ > - > > : < < - < . _ _ _ . > > > .
      human  . A A G A C U U C G G A U C U G G C G . A C A . C C C .
      mouse  a U A C A C U U C G G A U G - C A C C . A A A . G U G a
        orc  . A G G U C U U C - G C A C G G G C A g C C A c U U C .
             1       5         10        15        20        25    28
```

example structure:

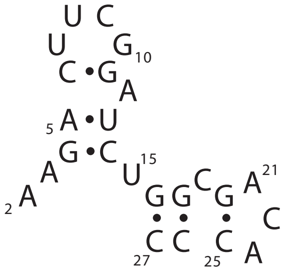

Supplement: S1 File — Includes alignments, and source code. (GZ) [file pcbi.1008387.s008.tar.gz › supplemental_material/src/rscape_v1.5.4.tar.gz!/rscape_v1.5.4/lib/infernal/infernal-1.1.2/documentation/userguide/Figures/input_alignment.pdf]
